# The Amazing Osteocyte

**DOI:** 10.1002/jbmr.320

**Published:** 2010-12-16

**Authors:** Lynda F Bonewald

**Affiliations:** Department of Oral Biology, University of Missouri–Kansas City Kansas City, MO, USA

**Keywords:** OSTEOCYTE, OSTEOCYTIC RESORPTION, REMODELING, OSTEOCYTIC OSTEOLYSIS

## Abstract

The last decade has provided a virtual explosion of data on the molecular biology and function of osteocytes. Far from being the “passive placeholder in bone,” this cell has been found to have numerous functions, such as acting as an orchestrator of bone remodeling through regulation of both osteoclast and osteoblast activity and also functioning as an endocrine cell. The osteocyte is a source of soluble factors not only to target cells on the bone surface but also to target distant organs, such as kidney, muscle, and other tissues. This cell plays a role in both phosphate metabolism and calcium availability and can remodel its perilacunar matrix. Osteocytes compose 90% to 95% of all bone cells in adult bone and are the longest lived bone cell, up to decades within their mineralized environment. As we age, these cells die, leaving behind empty lacunae that frequently micropetrose. In aged bone such as osteonecrotic bone, empty lacunae are associated with reduced remodeling. Inflammatory factors such as tumor necrosis factor and glucocorticoids used to treat inflammatory disease induce osteocyte cell death, but by different mechanisms with potentially different outcomes. Therefore, healthy, viable osteocytes are necessary for proper functionality of bone and other organs. © 2011 American Society for Bone and Mineral Research.

## Pioneers in the Study of Osteocytes

Before the introduction of PubMed and easy access to papers, some of the earliest observations regarding osteocytes, like the cells themselves, laid buried and difficult to find. My colleagues and I, like others, made assumptions that many of our observations were new and novel until these publications were made readily available. For example, over 100 years ago, it was postulated that osteocytes could remodel their extracellular matrix(1); over 40 years ago, it was thought that osteocytes were responsive to parathyroid hormone,(2) could remodel bone(3) and could express tartrate-specific acid phosphatase(4); and over 20 years ago, it was said that osteocytes were mechanosensory cells.(5) Marotti and Palumbo drew beautiful diagrams to illustrate their theories regarding osteocyte function and communication.(6) Histology was the major tool used by these early pioneers to generate their theories. Peter Nijweide was the first to isolate avian osteocytes.(7) Some of the earliest videos of bone cells including osteocytes were recorded by Kumegawa and colleagues.(8) With new technology, such as molecular and transgenic approaches, imaging, cell lines, systems biology, advanced instrumentation, and others, a dramatic increase in information on osteocyte biology has occurred in the last decade, leading to validation of old theories and the generation of new ones. These are highlighted in this review.

## Osteocytes as Descendants of Osteoblasts

**Fig. 1:**
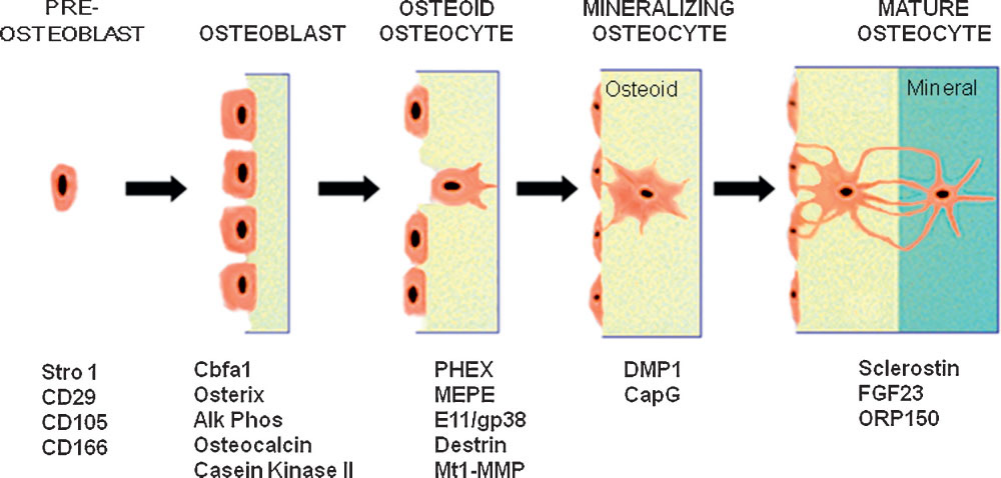
Expression of markers during osteoblast-to-osteocyte ontogeny. The osteocyte appears to be the descendant of the matrix-producing osteoblast, which is a descendant of the mesenchymal stem cell known to express markers such as Stro1, CD29, CD105, CD166. Matrix-producing osteoblasts express Cbfa1 and Osterix, necessary for osteoblast differentiation, followed by alkaline phosphase and collagen, necessary for the production of osteoid. Osteocalcin is produced by the late osteoblast and continues to be expressed by the osteocyte. By some unknown mechanism, some designated cells begin to embed in osteoid and begin to extend dendritic projections, keeping connections with already embedded cells and cells on the bone surface. Molecules such as E11/gp38 and MT1-MMP appear to play a role in dendrite/canaliculi formation, whereas molecules such as destrin and CapG regulate the cytoskeleton. PHEX, MEPE, and DMP-1 regulate biomineralization and mineral metabolism, and FGF-23 regulates renal phosphate excretion. FGF-23 is elevated not only in osteocytes from hypophosphatemic animals but also in those of normal rats.(112) Sclerostin is a marker of the mature osteocyte and is a negative regulator of bone formation.(45) ORP150 may preserve viability of this cell in a hypoxic environment.(23)

The *osteocyte*, defined as a cell located within the bone matrix, is descended from mesenchymal stem cells through osteoblast differentiation (Fig. 1). It was proposed by Manolagas(9) that the matrix-producing osteoblast either can become an osteocyte, a lining cell, or can undergo programmed cell death. His theories were based on the observations of another very early pioneer, Michael Parfitt, who proposed that osteoblasts must die by apoptosis.(10) Osteocytogenesis has been thought to be a passive process whereby a subpopulation of osteoblasts becomes passively encased in osteoid that passively mineralizes. It was theorized that an osteoblast-producing matrix/osteoid becomes trapped when its neighbor osteoblasts place osteoid on top of the embedding cell.(11) However, there are several arguments against osteocytogenesis being a passive process.

One of the first changes to take place in the embedding cell is the formation of dendritic processes. The cell undergoes a dramatic transformation from a polygonal cell to a cell extending dendrites toward the mineralizing front, which is followed by dendrites extending to either the vascular space or bone surface. The cell, once embedded in bone, especially cortical bone, has a polarity, especially with regard to directionality of mineral formation. The osteoid osteocyte must do two major functions simultaneously: regulate mineralization and form connective dendritic processes. The osteoid osteocyte can control and regulate mineralization,(12) and Holmbeck and colleagues(13) have shown osteocytogenesis to be an active invasive process requiring cleavage of collagen and potentially other matrix molecules. Osteocytes in mice null for the metalloproteinase MT1-MMP have significantly reduced number and length of dendritic processes. MT1-MMP is a membrane-anchored proteinase that can cleave collagens type I, II, and III, fibrin, fibronectin, and other matrix molecules. In this mouse model, the almost complete lack of dendritic processes did not appear to affect viability or density of osteocytes in contrast to studies by Zhao and coworkers,(14) where osteocytes in a mouse model of collagenase-resistant type I collagen did show increased apoptosis. However, it was impossible to determine the effect of a lack of dendritic processes on either osteocyte function or the effect(s) on the skeleton because the *MT1-MMP* null mouse exhibits multiple defects such as dwarfism owing to a lack of MT1-MMP in other skeletal tissues.(15)

**Fig. 2:**
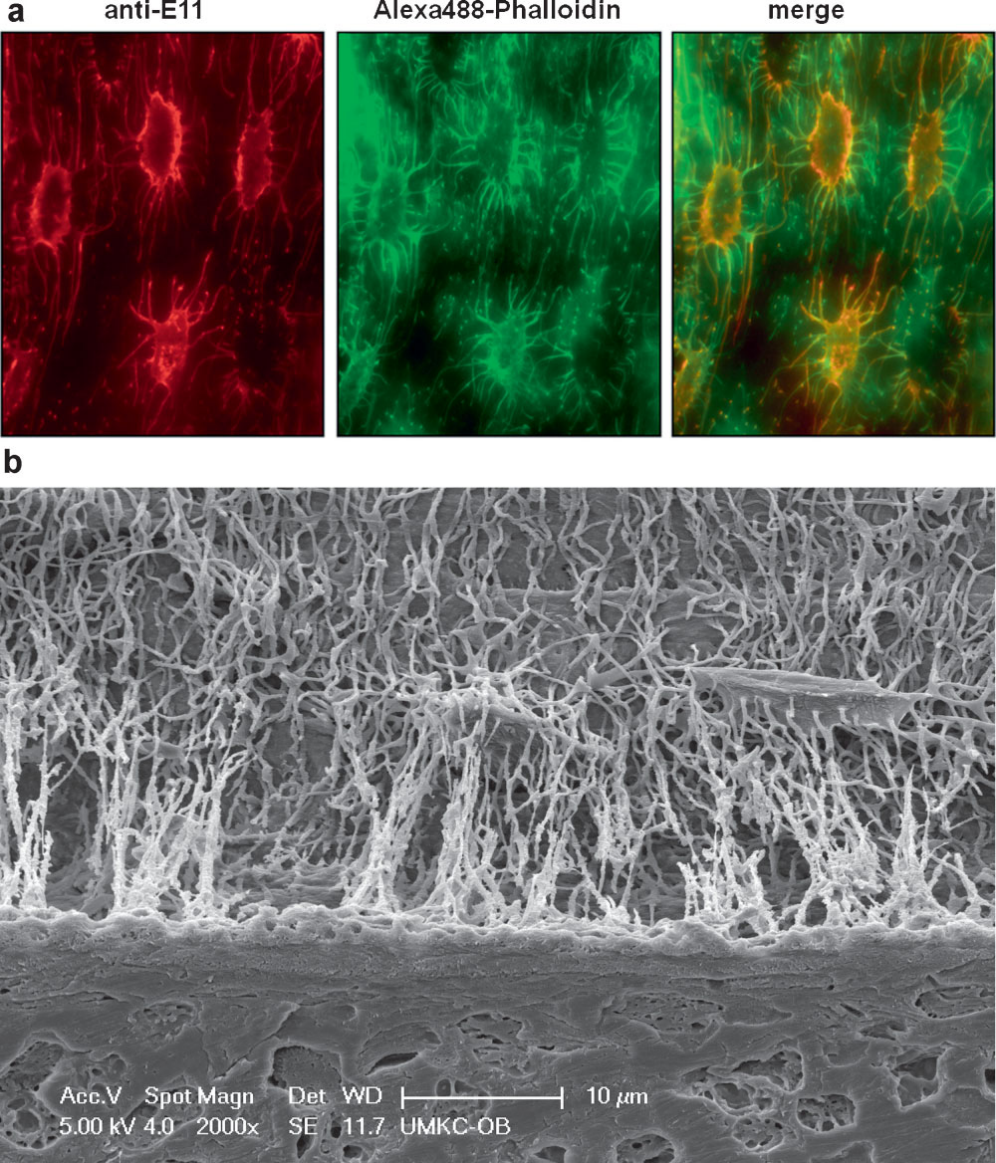
Visualization of early, embedding osteocytes. Using anti-E11 immunostaining and visualization of the actin cytoskeleton by alexa488 staining for phalloidin, one can visualize the embedding osteocyte and the early osteocyte in 12-day murine calvaria. The merged image shows that the majority of the E11 is on the cell surface and along the dendritic processes. Also, if one looks closely, the dendrites that end on the cell surface have a bulbous tip of unknown function. This structure must interface with the cells on the bone surface. The image is provided by Dr Sarah Dallas, University of Missouri at Kansas City. The second image is of an acid-etched resin-embedded murine sample showing an osteocyte lacuna sending canaliculi to the bone surface. Note the rough surfaces of canaliculi toward the bone surface and the smooth surface of canaliculi that project away from the bone surface, suggesting a difference between forming and formed canaliculi. Both sets of images demonstrate the complexity of this network and the interface of osteocytes with the bone surface.

Osteocyte morphology may be controlled by E11/gp38/podoplanin, a marker for the embedding osteoid osteocyte (Fig. 2). E11, also called *podoplanin*, *OTS-8*, *gp38*, or *PA2.25*, was first detected on the cell surface of osteocytes in rat bone(16,17) and odontoblasts in rat tooth.(18) It is also expressed in type I cells of rat lung and other tissues of brain, kidney, the lymphatic system, and skin. Application of fluid-flow shear stress on osteocyte-like MLO-Y4 cells increased the number and length of dendrites and was blocked by small interfering RNA against E11/gp38.(19) Conditional deletion in bone cells in vivo resulted in decreased canaliculi and increased trabecular bone.(20) In addition to E11, organized expression of tubulin, vimentin, and actin in cell bodies and dendrites of osteocytes is crucial to maintain their dendritic morphology.(21) Differences in distribution of fimbrin, villin, filamin, and spectrin, all actin-binding proteins, have been described accompanying the differentiation of osteoblasts to osteocytes,(22) as well as CapG and destrin, molecules necessary for cytoskeletal rearrangement.(23) Intriguingly, two groups of investigators have shown an increase in number of dendritic processes with skeletal age, suggesting that the already embedded osteocyte can either generate new processes or, alternatively, the newly embedded cells have a greater number of dendrites.(13,24)

As the osteoblast transitions to an osteocyte, alkaline phosphatase is reduced, and casein kinase II is elevated, as is osteocalcin.(25) Additional markers are expressed, including phosphate-regulating gene with homologies to endopeptidases on the X chromosome (PHEX), matrix extracellular phosphoglycoprotein (MEPE), dentin matrix protein 1 (DMP-1), fibroblast growth factor 23, (FGF-23), sclerostin, and ORP150, a factor thought to protect against hypoxia. The function of these molecules will be discussed below. Surprisingly, osteocytes also can express markers of osteoclasts, such as acid phosphatase and cathepsin K, under certain conditions such as lactation to remodel their perilacunar matrix.(26) These markers, as discussed in this review, show the versatility of this bone cell.

## Osteocytes as Inducers of Osteoclast Activation

The earliest data supporting the concept that osteocytes can send signals of bone resorption were gathered using isolated avian osteocytes showing support of osteoclast formation and activation in the absence of any osteotropic factors.(27) These observations were later duplicated using the osteocyte-like cell line MLO-Y4.(28) RANK ligand (RANKL) expression on dendritic processes appeared to be responsible. Conditioned medium from the MLO-Y4 cells also supports osteoblast differentiation(29) and, surprisingly, mesenchymal stem cell differentiation,(30) supporting the theory that osteocytes could be orchestrators of bone remodeling.

**Fig. 3:**
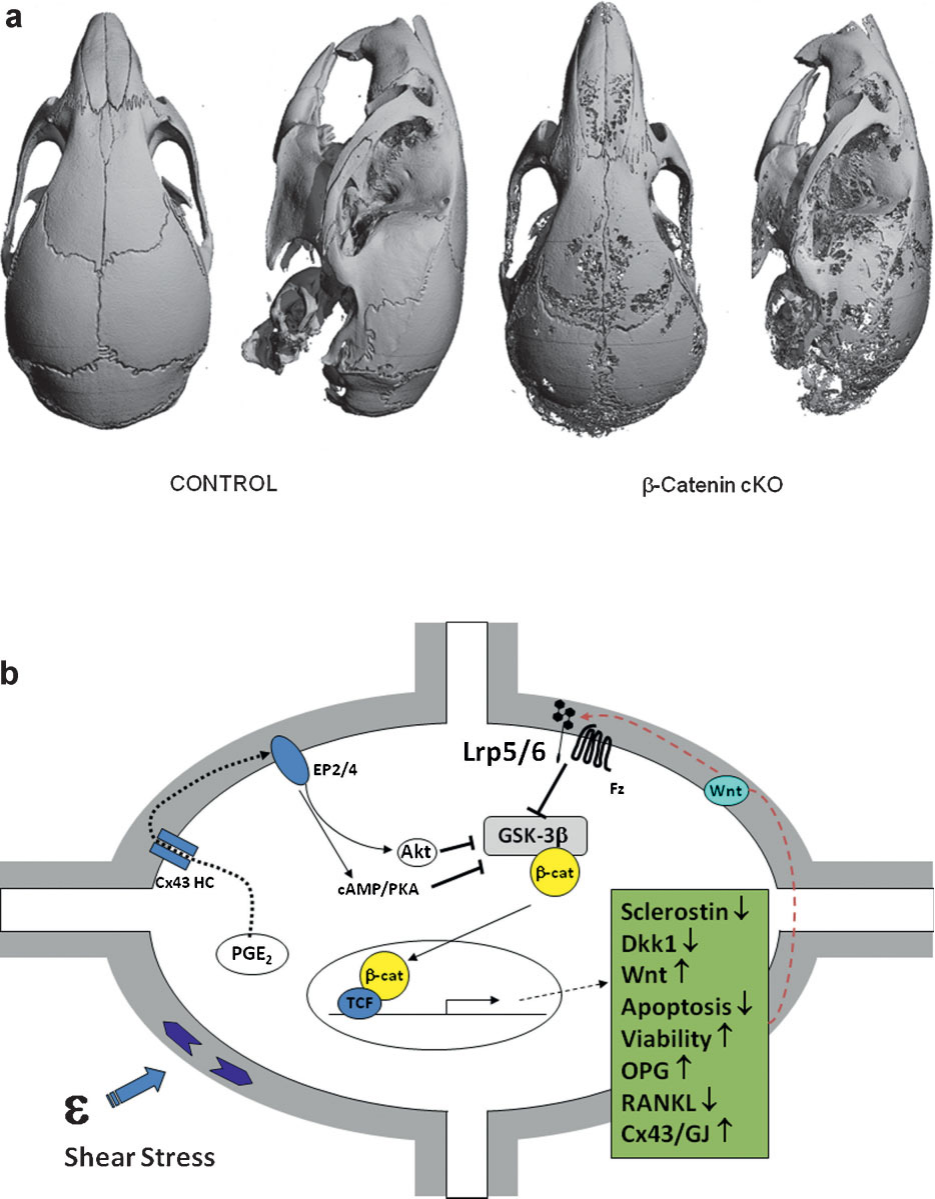
The Wnt/β-catenin pathway plays an important role in osteocyte function and viability and the maintenance of normal bone. Deletion of β-catenin in osteocytes results in bone with a “moth-eaten appearance.” The calvaria shown are from 14-week-old female control and conditional knockout (cKO) mice in which *β-catenin* is deleted using a Dmp1-Cre. The long bones from these animals show even greater porosity and fragility, thought to be responsible for death of these transgenic animals at an early age.(34) This bone porosity was due to increased osteoclast number and activity most likely owing to reduced expression of osteoprotegerin and an increase in RANKL, both found to be expressed in osteocytes. These observations support the role of β-catenin in osteocyte viability. The diagram integrates a number of observations regarding how mechanical loading in the form of fluid-flow shear stress regulates osteocyte viability,(40) function.(34) and communication(59) through the Wnt/β-catenin pathway. The unique triggering or crosstalk between prostaglandin and this pathway has been detailed previously.(58)

Both healthy and dying osteocytes can recruit osteoclasts to sites of remodeling. Osteocyte apoptosis can occur at sites of microdamage. Proapoptotic molecules are elevated in osteocytes immediately at the microcrack locus, whereas antiapoptotic molecules are expressed 1 to 2 mm from the microcrack,(31) showing that some osteocytes have protective mechanisms against apoptosis. Apoptotic osteocytes release apoptotic bodies expressing RANKL to recruit osteoclasts.(32) There are different forms of “dying,” such as apoptosis and necrosis, and osteocytes in these states may send different signals. Targeted ablation of osteocytes through necrosis was performed using the 10-kb Dmp1 promoter to drive the diptheria toxin receptor expression in osteocytes.(33) Injection of a single dose of diphtheria toxin eliminated approximately 70% of osteocytes in cortical bone and generated osteoclast activation in these mice. Another model of defective osteocytes was the deletion of β-catenin in osteocytes(34) (Fig. 3). Clearly β-catenin is necessary for normal osteocyte function, and deletion results is an increase in osteoclast activity and the generation of a porous bone phenotype. Osteocyte cell death and cell survival are reviewed below.

## The Importance of Osteocyte Death

Osteocyte cell death can occur in association with such pathologic conditions as osteoporosis and osteoarthritis, leading to increased skeletal fragility.(35) Such fragility is considered to be due to loss of the ability to sense microdamage and/or signal repair. Oxygen deprivation, such as occurs during immobilization, has been shown to promote osteocyte apoptosis,(36) as does glucocorticoid treatment(35) and withdrawal of estrogen.(37,38) Tumor necrosis factor α (TNF-α) and interleukin 1 (IL-1) have been reported to increase with estrogen deficiency and also induce osteocyte apoptosis (for review, see ref. (39)).

Inhibitors of osteocyte cell death include estrogen and selective estrogen receptor modulators, bisphosphonates, calcitonin, CD40 ligand, calbindin-D28k, and monocyte chemotactic proteins 1 and 3 (MCP1 and MCP3; for review, see ref. (39)). Recently, mechanical loading in the form of fluid-flow shear stress to mimic bone fluid flow in the osteocyte lacunocanalicular network has been shown to block glucocorticoid-induced apoptosis.(40) This was shown to be mediated through the release of prostaglandin, which activated the Wnt/β-catenin pathway. Alternatively, it also has been postulated that the reason for the opposite effects of mechanical loading and glucocorticoid on apoptosis of osteocytes is their opposing actions on the family of focal adhesion kinases (FAKs) and the proline-rich tyrosine kinase 2 (Pyk2). These investigators used substrate stretching as a form of mechanical loading to prevent apoptosis and showed activation of FAKs and extracellular signals–regulated kinases (ERKs).(41) It also was proposed that glucocorticoids oppose FAK/ERK signaling through Pyk2 and c-Jun N-terminal kinase (JNK)(42) and that extracellular matrix (ECM)–integrin/FAK signaling is linked with the the Wnt/β-catenin pathway.(43) Therefore, whether mechanical loading is applied through fluid-flow shear stress or through substrate stretching, the Wnt/β-catenin pathway appears to be involved.

In addition to undergoing programmed cell death, osteocytes can undergo a process of self-preservation called *autophagy*, especially in response to glucocorticoid.(44) Autophagy is a lysosomal degradation process necessary for recycling of cellular products. During autophagy, parts of the cytoplasm and intracellular organelles are localized within autophagic vacuoles for delivery to lysosomes for degradation. Autophagy has been proposed as a “double-edged sword” that can protect cells from apoptosis but, on the other hand, can destroy cellular components. Autophagy can preserve viability or, alternatively, can be a self-destructive process that leads to cell death. Therefore, in addition to responding to external agents or stimuli with necrosis and apoptosis, osteocytes can undergo autophagy to preserve self until favorable conditions return.

Osteocyte viability clearly plays a significant role in the maintenance of bone homeostasis and integrity. However, whereas blocking osteocyte apoptosis may improve diseases such as bone loss owing to aging or to glucocorticoid therapy, osteocyte apoptosis may be essential for damage repair and normal skeletal replacement. Any agents that block this process may exacerbate conditions in which repair is required.

## The Role of the Wnt/β-Catenin Pathway in Osteocyte Function and Viability

Negative regulators of the Wnt/β-catenin pathway such as Dkk1 and sclerostin are highly expressed in osteocytes. Dkk1 is expressed throughout the body, but sclerostin is expressed mainly in osteocytes. Mature osteocytes have been shown to produce sclerostin, coded by the gene *SOST*, that can inhibit osteoblast activity.(45) Downregulation of Dkk1 and SOST may create a permissive environment in which Wnt proteins already present can activate the Wnt pathway. Mutations of *SOST* causes high bone mass in humans,(46) as does deletion in mice.(47) Sclerostin was thought to be a bone morphogenetic protein (BMP) antagonist but later was discovered to be involved in the Wnt pathway as an antagonist against lipoprotein receptor 5 (LRP5), a positive regulator of bone mass.(48,49) Mechanical loading also has been reported to reduce sclerostin expression,(50) as does parathyroid hormone (PTH), which may account for some of the anabolic effect of PTH on bone formation.(51,52) PTH also appears to reduce sclerostin levels in patients.(53) Bone response to unloading was proposed to be due to elevated sclerostin.(50,54) Antibody to sclerostin is being considered as a new drug against postmenopausal osteoporosis(55,56) because of its specificity and its anabolic effect on bone formation. Other potential uses include disuse-induced bone loss, bone repair, fracture healing, and accelerated implant fixation. This molecule, more than any other, has served to raise interest in the osteocyte.

Mechanical loading of MLO-Y4 cells by fluid-flow shear stress protects against dexamethasone-induced apoptosis, and the mechanism for this protective effect of mechanical loading appears to be partially mediated through prostaglandin E_2_ (PGE_2_) crosstalk with β-catenin signaling.(40) Both PGE_2_ and fluid-flow shear-stress treatment result in increased phosphorylation of GSK-3β and β-catenin nuclear translocation.(57) Shear stress, through PGE_2_ release, activates both PI3K/Akt and cAMP-PKA signaling, which converge to inactivate GSK-3, leading to the increase in nuclear accumulation of β-catenin. Therefore, the Wnt/β-catenin signaling pathway plays a role not only in bone response to loading but also in osteocyte apoptosis.(58) β-Catenin also has been shown to bind to the Cx43 promoter, stimulating Cx43 expression and functional gap junctions between osteocytes.(59) Therefore, in addition to playing a role in osteocyte viability in response to shear stress, the β-catenin pathway is important in osteocyte communication (Fig. 3).

Other signaling pathways are activated in response to mechanical loading and also may cross talk with the Wnt/β-catenin signaling pathway. The estrogen receptor α isoform (ERα) may play a role in shuttling β-catenin into the nucleus in response to mechanical strain in osteoblasts.(60) This may explain in part how estrogen regulates bone mass through crosstalk between ERα with Wnt/β-catenin signaling. Therefore, multiple signaling pathways such as the estrogen and prostaglandin signaling pathways may converge through β-catenin.

As stated earlier, targeted deletion of β-catenin in osteocytes results in a bone phenotype with a “moth-eaten appearance,” clearly showing the importance of this pathway in the maintenance of normal bone(34) (Fig. 3). These published studies showed that osteocyte β*-*catenin is required for the expression of the antiosteoclastogenic factor osteoprotegerin (OPG) in osteocytes. Surprisingly, it was found that osteocytes express RANKL and OPG at levels comparable with or exceeding those of osteoblasts. These data support previous findings that osteocytes recruit osteoclasts and that osteocytes are key regulators of bone homeostasis.

## Osteocytes as a Source of Factors and Regulators of Mineral Metabolism

Osteocyte-specific or highly selective proteins have been shown to function in mineral metabolism. One of these is sclerostin, which was described earlier. Osteocytes appear to also regulate phosphate and biomineralization through molecules such as PHEX, DMP-1, MEPE, and FGF-23.(61) All of these are highly expressed in osteocytes.(62–64) Autosomal recessive hypophosphatemic rickets in patients is due to mutations in *DMP1.*(65) *Dmp1* null mice have a similar phenotype to *Hyp* mice carrying a *Phex* mutation, that of osteomalacia and rickets owing to elevated FGF-23 levels in osteocytes.(64,65) Both *Dmp1* and *Phex* appear to downregulate FGF-23 expression, which, in turn, allows reabsorption of phosphate by the kidney, thereby maintaining sufficient circulating phosphate to maintain normal bone mineral content. In the absence of either *Dmp1* or *Phex*, FGF-23 is elevated in the osteocyte and in the circulation, leading to phosphate excretion by the kidney, thereby reducing circulating phosphate responsible for osteomalacia and rickets. Based on these observations, my colleagues and I proposed that the osteocyte lacunocanalicular network can function as an endocrine system, targeting distant organs such as kidney.(65)

FGF-23 may be more than just a regulator of renal phosphate handling.(66) In addition to hypophosphatemic rickets, FGF-23 is elevated in osteocytes in patients with chronic kidney disease (CKD).(67) Progression in CKD is associated with elevated FGF-23.(68) Also, increased death with cardiovascular disease is associated with elevated FGF-23, as is calcification.(69) It will be important to determine if this osteocyte-derived factor has a direct effect on tissues other than the kidney. The unraveling of these interactions should lead to insight into diseases of both hyper- and hypophosphatemia, in addition to other diseases such as CKD and cardiovascular disease.

## Osteocytic Remodeling of the Perilacunar Matrix

Since the surface area of the osteocyte lacunocanalicular system within bone is several orders of magnitude greater than the area of the bone surface,(70) removal of only a few angstroms of mineral per osteocyte would have significant effects on circulating, systemic ion levels. Belanger coined the term *osteocytic osteolysis* and suggested that either PTH or low-calcium diet can induce this function in osteocytes.(2,3) Enlarged lacunae were found in bone from renal osteodystrophy patients,(71) in rats sent into space,(72) and in alveolar bone of hibernating ground squirrels.(73) Periosteocytic lesions in patients with X-linked hypophosphatemic rickets have been described,(74) as has a sphere of hypomineralized matrix around lacunae in prednisolone-treated rats.(75) Changes in properties of perilacunar bone matrix and lacunar size would influence fracture risk, and any mechanism that changes the material properties of the perilacunar matrix will have consequences on mechanosensation by osteocytes.(76,77)

Conversely, in 1971, Baylink showed tetracycline binding to the osteocyte perilacunar matrix, which led him to suggest that osteocytes have the ability to form bone.(4) Later, Zambonin-Zallone and coworkers used the egg-laying hen and several methods, including autoradiography and tetracycline labeling, to show that at least 20% of the osteocytes are active in bone formation.(78) Both these early observations support the hypothesis that osteocytes can form new perilacunar matrix.

During lactation, osteocyte lacunae are enlarged significantly in both cortical and trabecular bone compared with virgin and postweaned animals,(79) showing that *healthy* osteocytes can remove and replace their perilacunar matrix and potentially play a role in mineral homeostasis during a calcium-demanding condition such as lactation. Additionally, the widths of canaliculi were found to be significantly increased. Gene array analysis of osteocytes from lactating animals revealed an elevation of genes products known to be used by osteoclasts to remove bone, including tartrate-resistant acid phosphatase, cathepsin K, and others, and these returned to levels similar to virgin gene expression levels on weaning. These observations suggest that the healthy osteocyte can both add and remove mineral from its lacunae and canaliculi. Therefore, the osteocyte is a unique cell expressing genes of both the mesenchymal and hemapoietic lineage. This remodeling was mediated through the PTH type 1 receptor because lactating animals lacking this gene in osteocytes failed to remodel their perilacunar matrix.(26) Targeted expression of constitutively active PTH type 1 receptor in osteocytes results in elevated bone remodeling and elevated bone mass.(80) Therefore, the PTH type 1 receptor most likely plays an important role is osteocyte viability and function.

In contrast to the young, healthy osteocyte within a hypomineralized matrix, the aging osteocyte may be subjected to hypermineralization of its perilacunar matrix,(81) and micropetrosis has been described in aging bone, where the lacunae fill in with mineral. This hypermineralization may lead to cell death with age, which, in turn, leads to the filling in of the lacunae with mineral. This would change the dynamics of bone fluid flow through the osteocyte lacunocanalicular network dramatically, potentially affecting osteocyte function and viability.

## Osteocytes as Mechanosensory Cells

Another early postulated function for osteocytes is as a mechanosensory cell because of their location in bone and their complex dendritic network. The lining cell also has been hypothesized to be a major mechanosensory cell in the adult skeleton, but little is known about this type of bone cell. In vivo, the ideal frequency, intensity, and timing of loading are known that will increase bone mass.(82–84) Whereas parameters of in vivo loading are well characterized, a major challenge has been to identify in vitro experiments that replicate in vivo results. The fact that mechanical loading and unloading change osteocyte gene expression in vivo shows that load affects osteocyte function.(50,85–87)

Little is known about the bone fluid that flows through the osteocyte lacunocanalicular system, except that a molecular weight/mass cutoff of 70 kDa, the size of bovine serum albumin (BSA), exists.(88) Injection of dye into the tail vein of a mouse results in complete diffusion through the lacunocanalicular system within minutes. It has been proposed that bone fluid flow is driven by extravascular pressure as well as applied cyclic mechanical loading of osteocytes and that the peak physiologic loads are in the range of 8 to 30 dyn/cm^2^.(89) Whereas bone loss owing to hind limb unloading is restored with restored blood flow,(90) blood pressure does not play a role in regulating bone mass.(91) Recently, real-time measurement of load-induced solute transport has been shown, suggesting a peak shear stress on osteocyte processes of 5 Pa.(92) However, there are still many unanswered questions with regard to magnitude, frequency, and type of flow, such as pulsatile or oscillatory, to which the osteocyte may be subjected.

Early in vitro experiments used hydrostatic pressure and substrate stretching, whereas now fluid-flow shear stress is used because primary osteocytes are more sensitive than osteoblasts and more sensitive to shear stress than to substrate stretching.(93,94) MLO-Y4 osteocyte-like cells are several orders of magnitude more sensitive to fluid-flow shear stress with regard to release of prostaglandin than 2T3 osteoblast-like cells.(57) Shear stress has many effects on MLO-Y4 cells, including the release of nitric oxide, adenosine triphosphate (ATP), and prostaglandins; opening of hemichannels and gap junctions; promotion of dendrite elongation; bending of cilia; prevention of apoptosis; initiation of signaling pathways such as the Wnt/β-catenin, protein kinase A (PKA), and other signaling pathways; induction of β-catenin translocation to the nucleus; activation of gene transcription and translation; etc.—and the list continues to grow. To validate observations using cell lines, primary osteocytes can be prepared by sequential alternating collagenase digestions with EDTA,(7,95) but the yields continue to be low, especially with increasing age. Mice in which the 8-kb *Dmp1* promoter, driving green fluorescent protein (GFP) expression in osteocytes, is active have been used to obtain a highly purified GFP-positive population.(96) New transgenics are being made with labeled osteocytes such as the *Sost* promoter driving GFP, which will allow tracking of isolated mature osteocytes.

The osteocyte may have several means to sense load, such as through the cell body, the dendritic processes, or bending of cilia.(97) It has been proposed that the osteocyte senses load only through its processes(98) or through both the cell body and the processes.(76) Recently, it has been shown that glycocalyces on the surfaces of dendritic processes, but not on the cell body, play an essential role in mechanotransduction, whereas another, different mechanosensing mechanism is active on the cell body.(99) It also has been proposed that the osteocyte senses load through cilia, single flagellar-like structures found on every cell.(100,101) Unlike kidney cells, cilia in bone cells do not mediate calcium flux in response to fluid flow but do induce the release of prostaglandin.(101) Mice with impaired polycystin 1 (PC-1) function develop osteopenia owing to impaired osteoblast-mediated bone formation.(100) Mice with conditional deletion of *Pkd1* were developed using DMP-1-Cre, and such mice are smaller than controls at 1 month, but only minor differences were observed in 16-week-old mice, showing a recovery of bone mass. However, a dramatic decrease in response to anabolic loading was observed.(102) This shows that PC-1 in osteocytes is essential for the bone anabolic response to load.

Rapid osteocyte signals in response to shear stress include release of nitric oxide (NO), ATP, and prostaglandin. Just deleting only one of the three rapidly released small molecules will inhibit the bone anabolic response to loading. NO is a mechanical mediator that appears to be released around the same time as PGE_2_ from osteocytes,(93) and endothelial NO synthase is found in osteocytes.(103) In bone, NO inhibits resorption and promotes bone formation. Both osteoblasts and osteocytes release NO in response to mechanical strain or fluid-flow shear stress.(104) ATP and intracellular calcium also can be released from osteocytes in response to extracellular calcium or mechanical stimulation.(105,106) The P2X7 nucleotide receptor, an ATP-gated ion channel expressed in many cell types, may play a role in mechanosensation because deletion resulted in a 70% reduction in bone anabolic response in mice.(107) Blockers of P2X7 receptors suppressed prostaglandin release, whereas agonists enhanced release in bone cells, suggesting that the P2X7 receptor is necessary for release of prostaglandin in response to mechanical load.

In vivo prostaglandin induces new bone formation, and indomethacin blocks the effects of anabolic loading.(108) Prostaglandin appears to be released through hemichannels, unopposed halves of gap-junction channels, in response to shear stress.(109) Hemichannels are opened through association with perturbed integrin in response to shear stress and the glycocalyces of the osteocyte dendritic processes are necessary for integrin attachment to hemichannels.(99) Hemichannels expressed in MLO-Y4 cells also function as transducers of the antiapoptotic effects of bisphosphonates.(110) Therefore, hemichannels in osteocytes have multiple functions, including the release of signaling factors and protection of cell viability (for review, see refs. (39) and (111)).

## What's Next?

Where will the next decade or two take us with regard to osteocyte biology and function? Will other osteocyte factors be discovered that can be used for drug targeting and potential therapeutics? Will additional factors be discovered that have targeted effects on distant organs? Bone frequently has been referred to as a storehouse of factors, and this usually refers to factors within the bone matrix. But what if the osteocyte lacunar network is a storehouse for regulatory factors rapidly released—unlike the bone matrix, which requires some degradation. Preserving the health and function of this network becomes imperative.
